# Systematic review and meta-analysis on patented and non-patented vitrification processes to ovarian tissue reported between 2000 and 2021

**DOI:** 10.1590/1984-3143-AR2023-0065

**Published:** 2023-11-10

**Authors:** Éverton Pimentel Ferreira Lopes, Gildas Mbemya Tetaping, Marco Aurélio Schiavo Novaes, Regiane Rodrigues dos Santos, Ana Paula Ribeiro Rodrigues

**Affiliations:** 1 Laboratório de Manipulação de Oócitos e Folículos Ovarianos Pré-Antrais (LAMOFOPA), Faculdade de Medicina Veterinária, Universidade Estadual do Ceará, Fortaleza, CE, Brasil; 2 Schothorst Feed Research, Lelystad, The Netherlands

**Keywords:** intellectual property, cryopreservation, ovarian follicles, fertility preservation

## Abstract

Due to the great interest in ovarian cryopreservation and, consequently conservation and restoration of female fertility in the last decades, different vitrification procedures (vitrification devices or solutions) have been developed, patented, and used both for academic research purposes and for clinical use. Therefore, the present study aimed to provide a systematic review and meta-analysis of data obtained from the application of different patented and non-patented vitrification devices and solutions in different countries. For this purpose, relevant observational studies published between the years 2000 to 2021 were selected to verify the efficiency of ovarian vitrification processes on parameters such as morphology, viability, and apoptosis in preantral ovarian follicles after transplantation or in vitro culture. Our research revealed that, although several countries were considered in the study, the United States and Japan were the countries that registered the most processes, and 22 and 16 vitrification devices and solutions out of a total of 51, respectively were patented. Sixty-two non-patented processes were also considered in the study in all countries. We also observed that transplantation and in vitro ovarian culture were the techniques predominantly used to evaluate the efficiency of the devices and vitrification solutions, respectively. In conclusion, this review showed that patented or non-patented protocols available in the literature are able to successfully preserve preantral follicles present in ovarian tissue. Despite the satisfactory results reported so far, adjustments in ovarian vitrification protocols in order to minimize cryoinjuries to the follicles remain one of the goals of cryopreservation and preservation of the female reproductive function. We found that vitrification alters the morphology and viability, and offers risks leading in some cases to follicular apoptosis. However, adjustments to current protocols to develop an optimal procedure can minimize damage by not compromising follicular development after vitrification/warming.

## Introduction

Patents represent the optimal points in the career of inventors, being a temporary property title granted by the state of law to its holder, who then has an exclusive right to commercially exploit a product, a manufacturing process, or improvement of existing products and processes (Kevles, 2007). A patent is a codification of an invention, containing a set of new information needed to build something new (Chitale et al., 2020). In this way, it is possible to go further with the conception that the patent is a way to encourage the process of technological evolution, making companies and individuals develop new technologies for their use in society (Huang et al., 2021). It is worth noting that in recent years, universities have also played a fundamental role in this process, since besides their two publicly and well-known missions (teaching and research), they now have a third mission, which is to develop society and the economy (Etzkowitz and Zhou, 2017).

In the context of assisted reproduction biotechnologies in animals or humans, it is pertinent to consider the development of protocols for the cryopreservation of germ cells, such as the thousands of pre-antral follicles present in the ovary. Cryopreservation of these follicles acts as auxiliary biotechnology to the various assisted reproduction techniques, contributing to the preservation/restoration of female fertility. Pre-antral follicles, especially the primordial, constitute the ovarian reserve, representing about 95% of the entire follicular population of the mammalian ovary (McGee and Hsueh, 2000). Thus, over the last two decades, in the human species, ovarian tissue cryopreservation has become the only viable option for young women who wish to safeguard their fertility before gonadotoxic therapies (Kristensen and Andersen, 2018). Ovary cryopreservation has also been an approach for the preservation of genetic material, aiming at the conservation of rare or endangered animals, as well as animals of high genetic, economic, or social value, ensuring the security of biodiversity. Ovarian follicles can be stored at cryogenic temperatures for indefinite periods. At the appropriate time, this material can be used (Kong et al., 2021) to restore female fertility and obtain a new generation of fertilizable oocytes from these follicles.

Studies involving different cryopreservation methods, solutions, or both, have been developed to define the most adequate or optimizing available protocols for the cryopreservation of ovaries, ovarian follicles, and oocytes. A key approach for cryopreservation of these structures is vitrification, an ultrarapid cooling technique. Vitrification is a well-established procedure, that avoids problems of ice nucleation and crystallization by bringing the sample up to liquid nitrogen temperature at a rate of ~ 10,000 to 50,000 °C/min (Yagoub et al., 2022). This method is attractive because it is generally a rapid and low-cost procedure and does not require special equipment such as programmable freezing machines. A wide variety of techniques for vitrification of ovarian tissue, involving different devices (Sugishita et al., 2021), types and concentrations (Gupta et al., 2022), and forms of exposure (Locatelli et al., 2019) to cryoprotective agents (CPAs) are available in the literature. Several research laboratories and clinics around the world have developed their protocols and obtained copyrights through patents and commercialized their cryogenic products (vitrification devices and solutions, for example). Nevertheless, several other devices, techniques, or protocols that have been effectively used were not subject to patenting, as we will explore subsequently.

After vitrification, the ovary can be transplanted (xeno or autotransplantation) to resume hormonal and reproductive functions in vivo, or it can be cultured in vitro, aiming at obtaining potentially fertilizable oocytes (Dolmans et al., 2020). Regarding transplantation, births have already been reported in humans (Suzuki et al., 2015), sheep (Bordes et al., 2005), and laboratory animals (Okamoto et al., 2018). Despite these advances, it is not clear which devices, solutions, and/or methods are the most indicated to preserve ovarian preantral follicles present in the mammalian ovary.

The objective of the present study was to provide a systematic review and meta-analysis of different methods of ovarian tissue vitrification, considering patented and non-patented cryoprotectant devices and solutions. For this, relevant observational cohort studies were included.

## Materials and methods

**Figure 1:**
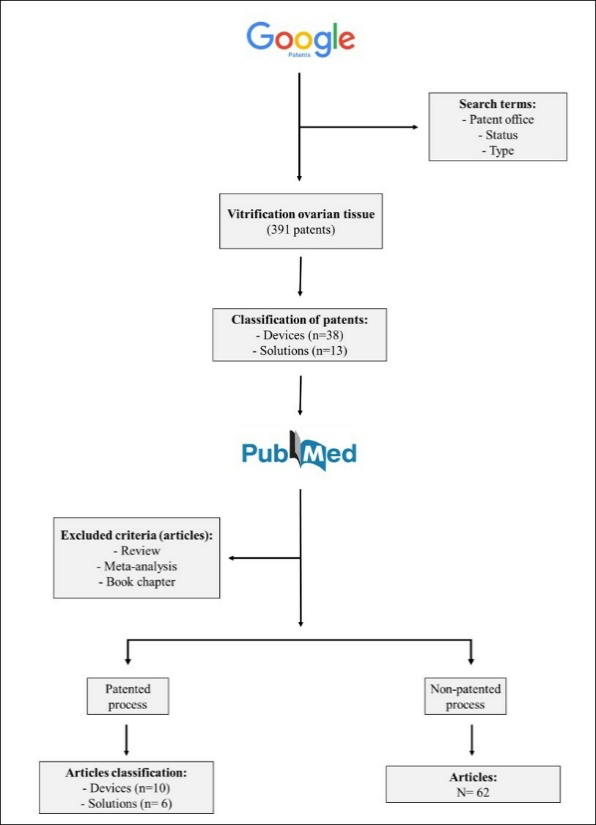
Schematic flowchart showing the steps of the present systematic review and meta-analysis on different ovarian tissue vitrification protocols.

**Figure 2:**
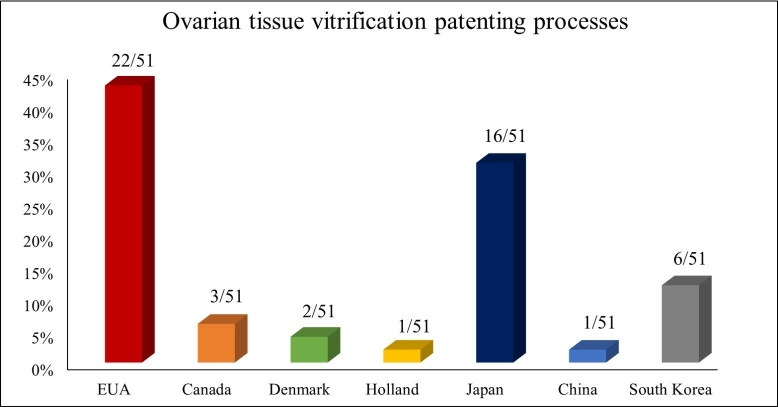
Percentage data of patented procedures on vitrification of ovarian tissue in different countries.

In the present study, as shown in Figure 1, we used the google patents repository as a search source to locate deposited patents. The chosen search term was “*Ovarian tissue vitrification*”. After extensive consultation, patents were selected with their grant status. In addition to the alignment of search terms, we selected the main countries that stand out in publications involving the search term. The countries selected were the United States of America, Canada, Brazil, Denmark, Holland, Japan, China, and South Korea, in which we found patents for vitrification devices and solutions for ovarian tissue (Figure 2).

The following electronic search strategy was performed in Google Patents, followed by a search in PubMed/ScienceDirect: (Ovarian tissue vitrification) and English [lang]). The published and peer-reviewed full-text articles identified from this search were evaluated by reviewing the titles and abstracts. A systematic review and meta-analysis of the available literature were performed for all relevant full-text articles (registered patents and no patent) published in PubMed (January 2000 until December 2021). Only manuscripts in English were considered to evaluate the development of methods to vitrify ovarian tissue.

Publications in the form of reviews, meta-analyses, or book chapters were excluded. In addition, the bibliographies of the included articles were reviewed for further studies. Two independent reviewers (Everton Pimentel and Gildas Mbemya) reviewed the included reports, as previously suggested by Zhou et al., 2016. Data from the text, graphs, and tables were analyzed. The patented studies (a total of 51 articles) were selected in the platform Google Patents and subsequently were divided into two main categories: vitrification devices (n=38), vitrification solutions (n=13), and no patented process (62 articles).

### Data extraction and statistical analysis

Data were independently assessed by two reviewers, grouped, and tabulated in Microsoft Excel 2016 program. The information extracted for data evaluation were as follows: year of publication, protocol (vitrification device and/or solution), technics (in vitro culture or transplantation), evaluated structure (follicles included in the ovarian tissue), and characteristics of the follicular population analyzed by morphology. Regarding morphology, the preantral follicles were classified as normal or degenerated and according to the degree of development as primordial, transitional, primary, and secondary. Data on morphology and viability of preantral follicles, as well as information on apoptosis in these structures, were grouped for meta-analysis according to recommendations (Higgins et al., 2013). For data analysis, we used Review Manager 5.4.1 software. The Mantel-Haenszel χ2 test and the I^2^ test were used to assess statistical heterogeneity. When the value of I2 was less than 50%, the heterogeneity was considered acceptable. In this case, a fixed effect model was used for calculations in the absence of evidence of heterogeneity; otherwise, a random effect model was applied. Odds ratios (OR) were used to evaluate the dichotomous variables, accompanied by 95% confidence intervals (CI). For these trials, a *P* value < 0.05 was considered statistically significant.

## Results

A total of 391 patents and 452 full-text articles were identified in the initial search. During the initial review of titles and abstracts of all 391 patents, 340 were excluded while the remaining 51 met our inclusion criteria and were evaluated (Figure 1). Among the 452 publications found, 77 were excluded while the remaining 265 met our inclusion criteria. Based on these findings, we performed a meta-analysis of the data from both patented and non-patented processes. For this, the rate of vitrification success of ovarian tissue considered the normal follicular morphology, viability and apoptosis rate.

## Patented processes

### Devices

**Figure 3:**
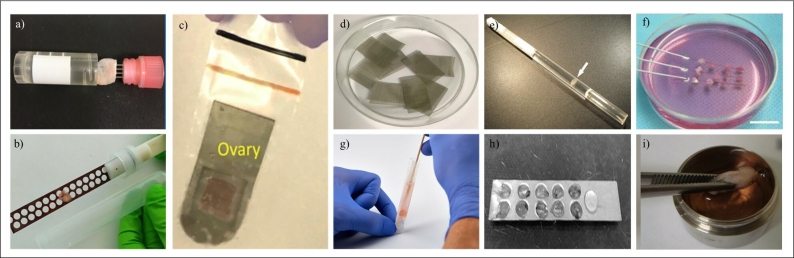
Illustrative images of devices used for vitrification of ovarian tissue. Patented devices: (a) Cryossuport ®, (b) Cryotissue ® e (c) Cryosheet ®. Non-patented devices: (d) stainless steel mesh, (e) straws, (f) acupuncture needle, (g) cryotubes, (h) aluminium foil, and (i) ovarian tissue cryosystem.

Vitrification refers to a physical process, in which the passage of a liquid solution occurs to form a glass-like state (Fuller and Paynter, 2004). Since the first description of the vitrification process (Luyet and Hodapp, 1938), several techniques and protocols have been formulated aiming to obtain an ideal vitrification solution that guarantees the maintenance of structures ensuring functionality after heating the biological material (Figure 3).

Regarding the vitrification of ovarian tissue, some factors should be considered, such as type, concentration, time, and temperature of exposure of the biological sample to intracellular cryoprotectant agents (CPA), as well as the device in which the biological material will be vitrified and stored in liquid nitrogen. Especially concerning the latter, the literature shows that different devices have been developed to ensure an adequate cooling rate of the biological sample uniformly, as well as to avoid contamination of the biological material by pathogens (Figure 3).

In the present review, 38 patents related to vitrification devices applied between the years 2001-2019 and active were highlighted (Table 1). The scientific articles that used the patented devices were accessible on the PubMed platform and were considered.

**Table 1:** Patents regarding vitrification device between the years 2001-2019.

**VITRIFICATION DEVICE**				
**Author**	**Patent ID**	**Woldwide applications**	**Publication**	**Device type**
Toner Mehmet	US6673607B2	2001	2004	uninformed
Robert Burghardt	US6521402B1	2001	2003	uninformed
Samuel Prien	US6615592B2	2001	2003	uninformed
Amir Arav	US6916602B2	2002	2005	open
Shuji Ueda	JP4359670B2	2004	2009	open
Amir Arav	US8580487B2	2005	2013	open
Naho Mi	JP4876922B2	2005	2012	closed
Noriko Kagawa	JP5224703B2	2007	2013	open
Amr Kader	US8030063B2	2009	2011	closed
Michael Cecchi	US9169460B2	2009	2015	open
Tomas Stojanov	DK2257155T3	2009	2019	closed
Nao Suzuki	JP5825571B2	2011	2015	closed
Anne Pelle Meddahi	US9521839B2	2013	2016	uninformed
Nao Suzuki	JP6438887B2	2013	2018	open
Meddy Emperor	JP6095689B2	2013	2017	uninformed
Atsushi Matsuzawa	US10492487B2	2014	2019	open
Atsushi Matsuzawa	US10039278B2	2014	2018	open
Kenji Momozawa	US10412958B2	2014	2019	open
Atsushi Matsuzawa	JP6124845B2	2014	2017	open
Kenji Momozawa	JP6022602B2	2014	2016	open
Susaki Live	JP6294729B2	2014	2018	open
Hatsushi Matsuza	KR101883813B1	2014	2018	open
Atsushima Tsuza	KR101988140B1	2014	2019	open
Nao Suzuki	US10561141B2	2015	2020	closed
Samuel Kim	US9228925B2	2015	2016	closed
Atsushi Matsuzawa	US10624335B2	2015	2020	open
James Benson	CA2943819C	2015	2017	closed
Nao Suzuki	JP6592004B2	2015	2019	closed
Nao Suzuki	JP6686267B2	2015	2020	closed
Atsushi Matsuzawa	JP6230582B2	2015	2017	open
Atsushi Matsuzawa	JP3202359U	2015	2016	open
Atsushi Matsuzawa	JP3202440U	2015	2016	open
Atsushima Tsuza	KR101962052B1	2015	2019	open
Pravansu Mohanty	US10568318B2	2015	2020	open
James Benson	US9936690B2	2015	2018	closed
Jiang Ligang	CN207305900U	2017	2018	closed
Sho Yoshida	JP6768955B2	2018	2020	open
Lee Jang	KR102146379B1	2019	2020	open

ID: Identification.

**Figure 4:**
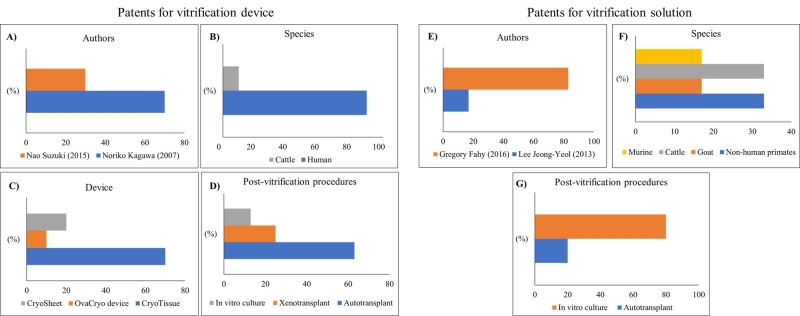
Percentage data of patents for vitrification devices (A-D) and solutions (E-G). (A) authors, (B) investigated species, (C) patented vitrification devices, (D) procedures applied after vitrification, (E) authors, (F) investigated species, and (G) procedures applied after vitrification, extracted from the selected studies in this review.

Based on our findings, considering the 38 patents on vitrification devices for ovarian tissue, it was possible to access only 11 articles published in scientific journals. Figure 4 shows respectively, the main authors, species studied, devices developed, and the procedures applied to evaluate follicular development after ovarian vitrification. In vitro culture was applied in 13% of the studies, while auto and xenotransplantation were employed in 62% and 25%, respectively.

Due to insufficient data on the morphology (normal and degenerated follicles) of preantral follicles after vitrification using the patented devices, it was not possible to perform the meta-analysis for this parameter. However, the data reported no significant difference in the morphology of fresh (primordial: 88% and primary: 77%) and vitrified (primordial: 80% and primary: 52%) follicles after vitrification of human ovarian tissue (Zhao et al., 2019).

Similar to morphology, there was insufficient data on the viability of preantral follicles after vitrification using the patented devices, and it was not possible to perform a meta-analysis for this parameter. However, Kagawa et al. (2009) obtained approximately 90% of viable oocytes after the vitrification of follicles included in ovarian tissue in the bovine and human species, highlighting the effectiveness of the methodology employed for clinical use.

**Figure 5:**
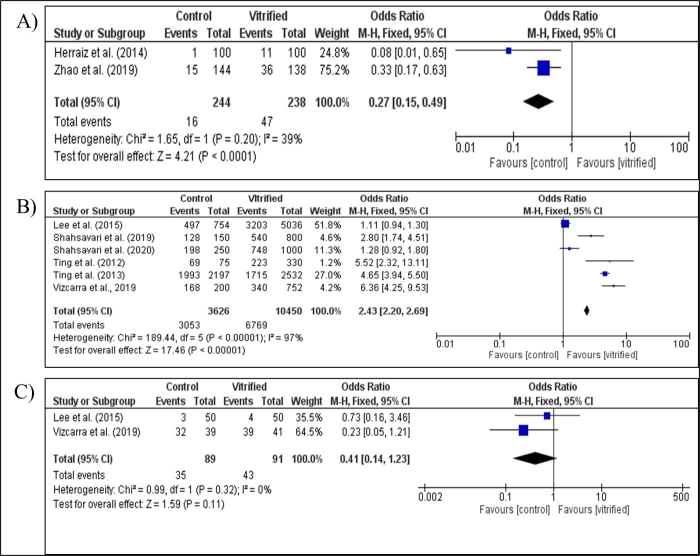
Meta-analysis on patented vitrification device and solutions. (A) Evaluation of apoptosis of preantral ovarian follicles after vitrification of ovarian tissue in vitrification devices. Evaluation of viability (B) and apoptosis (C) of preantral ovarian follicles after vitrification of ovarian tissue.

The TUNEL assay was used in two studies on patented devices (Herraiz et al., 2014; Zhao et al., 2019) for detecting follicular apoptosis through terminal end labeling of nucleic acids (Fig. 5A). Both studies indicated that vitrification induced a significant increase in apoptotic follicles compared to fresh follicles. There was significant evidence of heterogeneity between the two studies (I^2^ = 39%), and a fixed effect model was used for the grouped estimates. The pooled OR showed a significant difference in the proportion of apoptotic follicles after vitrification, demonstrating an approximately 4x higher chance of apoptosis after vitrification compared to the control (OR = 3.73; 95% CI 2.02 - 6.90; *P* = 0.00001).

### Vitrification solution

Thirteen active patents related to vitrification solutions were found between the years 1989 - 2017 as shown in Table 2. The scientific articles that used the patented solutions were accessible on the consulted platform were considered. Of the six studies investigated, four (80%) used in vitro culture, and one (20%) used autotransplantation as the post-vitrification procedure for follicular analysis. It is worth noting that only one (20%) study reported neither in vitro culture nor transplantation as a strategy for evaluating follicles post-vitrified (Figure 4).

**Table 2:** Patents regarding vitrification solution between the years 1989-2017.

**VITRIFICATION SOLUTION**				
**Inventor**	**ID**	**Woldwide applications**	**Publication**	**Status**
Thomas Glonek	CA1335723C	1989	1995	active
Boris Rubinsky	CA2074162C	1991	1999	active
Boris Rubinsky	US5358931A	1993	1994	active
Motomi Miyamoto	JP3694730B2	2000	2005	active
Gregory Fahy	CA2539274C	2004	2016	active
Meddahi Pelle	DK2804475T3	2013	2017	active
Hans Berg	KR102196974B1	2013	2020	active
Lee Jeong	KR101439231B1	2013	2014	active
Claudia Zylberberg	US10765111B1	2015	2020	active
Anthony Kennedy	US10448631B2	2016	2019	active
Bin-Ru She	US10815456B2	2017	2020	active
Lia Campbell	US10687525B2	2017	2020	active
Petrus Josephus	NL2019918B1	2017	2019	active

ID: Identification.

Of the thirteen patents available on vitrification solutions for ovarian tissue, it was possible to access six articles published in scientific journals referring to two patents. One publication for Lee Jeong-Yeol's registration (patent ID: KR101439231B1) and 5 publications for Gregory Fahy's registration (patent ID: CA2539274C). Lee Jeong's record (ID: KR101439231B1) was reported in a publication with mouse ovarian tissue (Lee et al., 2015). This patent refers to the addition of type III antifreeze proteins (AFP III) in the vitrification solution to improve the preservation of biological material at cryogenic temperatures (-196 ºC). Another additive in the vitrification solution was proposed by Gregory Fahy (CA2539274C). This author synthesized substances (synthetic polymers) with similar characteristics to antifreeze proteins. The synthetic polymers Supercool X-1000® and Supercool Z-1000® have been tested and reported in five publications on different species.

Preantral follicle viability was assessed in the six studies analyzed (Figure 5B), of which, four (bovine: (Shahsavari et al., 2019); non-human primates: (Ting et al., 2012, 2013); goat: (Vizcarra et al., 2020)) indicated that vitrification reduces the percentage of viable follicles compared to fresh follicles. The other two studies (mice: (Lee et al., 2015); bovine: (Shahsavari et al., 2020)) did not observe this association. There was significant evidence of heterogeneity among the six studies (I^2^ = 97%), so a random effect model was used for the pooled estimates. The pooled OR showed a significant reduction in the percentage of viable preantral follicles after vitrification (OR = 2.43; 95% CI 2.20 - 2.69; *P* = 0.00001).

Follicular apoptosis was evaluated in two studies (mice: (Lee et al., 2015); goat: (Vizcarra et al., 2020)), which indicated that there was no association of apoptosis between fresh and vitrified follicles (Fig. 5C). There was no significant evidence of heterogeneity between the two studies (I^2^ = 0%), and a fixed effect model was used for the grouped estimates. Similarly, the grouped OR showed no significant difference in the percentage of apoptosis after vitrification (OR = 0.41; 95% CI 0.14 - 1.23; *P* = 0.11).

### Non-patented processes

**Figure 6:**
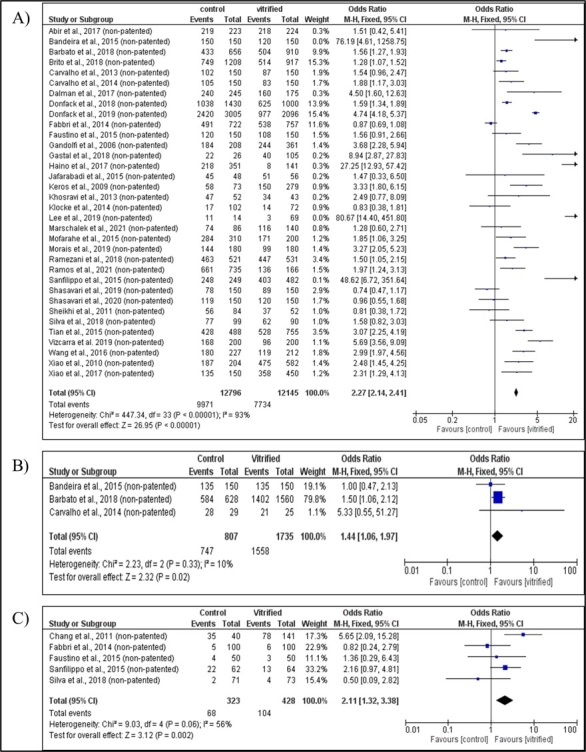
Meta-analysis of non-patented processes. Evaluation of the morphology (A), viability (B) and apoptosis (C) of preantral ovarian follicles after vitrification of ovarian tissue.

The morphology of preantral follicles was evaluated in 34 studies of different species (Figure6A) that used non-patented processes. Twelve (human: (Sheikhi et al., 2011; Khosravi et al., 2013; Fabbri et al., 2014; Klocke et al., 2014; Jafarabadi et al., 2015; Abir et al., 2016; Marschalek et al., 2021;); goat: (Carvalho et al., 2013; Faustino et al., 2015); bovine: (Shahsavari et al., 2019, 2020); ovine: (Silva et al., 2018)) observed no impairment of follicular morphology post-vitrification when compared to control. On the other hand, twenty-two studies (ovine: (Bandeira et al., 2015; Morais et al., 2019); goat: (Carvalho et al., 2014; Donfack et al., 2018, 2019; Montano Vizcarra et al., 2020); cat: (Brito et al., 2018); deer: (Gastal et al., 2018); human: (Gandolfi et al., 2006; Keros et al., 2009; Xiao et al., 2010, 2017; Mofarahe et al., 2015; Sanfilippo et al., 2015; Tian et al., 2015; Wang et al., 2016; Dalman et al., 2017; Barbato et al., 2018; Haino et al., 2018; Ramezani et al., 2018; Lee et al., 2019; Ramos et al., 2022) indicated that vitrification reduces the percentage of viable follicles compared to fresh follicles. Overall, there was heterogeneity (I^2^ = 93%) between the studies evaluated, so a random effect model was used for the pooled estimates. Considering the results, the pooled OR indicated a significant reduction in the percentage of viable preantral follicles after vitrification (OR=2.27; 95% CI 2.14 - 2.41; *P* = 0.00001).

Follicular viability was evaluated in three studies (Fig. 6B); two (Carvalho et al., 2014; Bandeira et al., 2015) did not observe an association between vitrification and follicular viability, however, the data presented by Barbato et al. (2018) indicated that vitrification reduces the percentage of viable follicles compared to fresh follicles. Overall, low heterogeneity (I^2^=10%) was observed among the evaluated studies, therefore, a random effect model was used for the pooled estimates. Considering the results, the pooled OR indicated a significant difference in the percentage of viable preantral follicles after vitrification (OR = 1.44; CI 95% 1.06-1.97; *P* = 0.02).

Cellular apoptosis was investigated in five studies (Fig. 6C); of these, four (Fabbri et al., 2014; Sanfilippo et al., 2015; Silva et al., 2018) did not indicate an association between vitrification and apoptosis. On the other hand, the study by Chang et al. (2011) indicated an association between increased apoptosis and vitrification. Heterogeneity was found among the studies evaluated (I^2^ = 56%), and a random effect model was used for grouped estimates. Overall, the grouped OR revealed a significant difference in the percentage of apoptotic follicles after vitrification, exhibiting an approximately 2x higher chance of apoptosis after vitrification compared to the control (OR = 2.11; 95% CI 1.32 - 3.38; *P* = 0.002).

## Discussion

To the best of our knowledge, this is the first article that brings to the attention of experienced and young scientists the development of patents related to devices and vitrification solutions for ovarian cryopreservation. We observe that countries like the United States of America and Japan, in addition to being pioneers and holders of more patents in this area, are also at the forefront regarding the filing of patents in relation to other countries in the world. As noted by other authors, this indicates a tendency for more economically developed countries to contribute to the dissemination of technological research and developments on a large scale. Despite this finding, several non-patented ovarian vitrification processes are described in the literature and can significantly contribute to the preservation of genetic potential and restoration of reproductive capacity and fertility in different species.

In humans, ovarian tissue cryopreservation is the only viable clinical option for young patients suffering from pathologies in the reproductive system or undergoing gonadotoxic treatments such as chemotherapy (Amorim et al., 2011; Dolmans et al., 2013). In animal species, this tool directly contributes to the implantation of germplasm banks, being fundamental to preserve the genetic biodiversity and the reproductive potential of rare and valuable animals (Santos et al., 2010; Comizzoli, 2017). In this regard, vitrification has been widely investigated as a method for ovarian cryopreservation, as it is widely used for the preservation of mammalian blastocysts and oocytes (Yagoub et al., 2022). In addition, it can reduce excessive ice crystal formation during the temperature-cooling process, minimizing the risk of mechanical damage to stromal and follicular cells (Leonel et al., 2019). Therefore, in this systematic review and meta-analysis, we address the efficiency of vitrification of ovarian tissue in 79 studies considering viability, morphology, and follicular apoptosis after vitrification using patented or non-patented processes (devices or vitrification solution).

To improve the available vitrification protocols and increase follicle survival and development rates after warming, many variations (vitrification devices or solutions) of this method have been reported. In human and bovine species, Kagawa et al., in 2015, developed the cryotissue (patent ID: JP5224703B2), an open device (that allows direct contact with liquid nitrogen) consisting of a metal strip specifically for housing ovarian tissue. Using this device, the authors obtained more than 89% viable oocytes from follicles present in the vitrified ovaries of both species. Another open device also patented was the CryoSupport or OvaCryo, developed by Suzuki and collaborators in 2015 (patent ID: JP6592004B2), consisting of four stainless steel needles inserted into the cap of a cryogenic tube, whose vitrification using such a device allowed to preserve the normal morphology of preantral follicles of non-human primates (Suzuki et al., 2012). Ovaries vitrified using this device were able to resume ovarian function after transplantation, resulting in the birth of two healthy children in humans (Suzuki et al., 2015).

According to the literature, the use of an open device for cryopreservation of biological material is still controversial, because, besides the risk of viral contamination (Porcu et al., 2021) some bacteria can survive at ultra-low temperatures (-196 ºC) such as that of liquid nitrogen (Marin et al., 2020). Thus, it is recommended that the cryopreservation and cryostorage process of valuable biological material be in devices that do not allow direct contact with liquid nitrogen (Hickman et al., 2020). In this sense, Suzuki and collaborators (2015) developed the Cryosheet, a closed device (without contact with liquid nitrogen) consisting of titanium, which improves thermal conduction and a portion of polypropylene attached to the edge for tissue fixation. The efficiency of this device was evaluated in human ovarian tissue considering the percentage of developing follicles (60%) and apoptosis (17%) (Sugishita et al., 2018, 2021). By avoiding contact of the ovary with possible dangerous pathogens present in the container or in the nitrogen itself, the use of Cryosheet or other closed devices, even if not patented, such as cryotubes, straws, etc.) are safer and therefore more recommended.

As highlighted in the results section, 60% of studies involving proprietary devices used ovarian autotransplantation after vitrification. In general, the ovarian tissue is transferred to the host and fixed in an environment or location similar to the original (orthotopic transplantation), aiming to resume endocrine and reproductive functions. Autotransplantation (grafting into the same individual ovary donor) has been widely used in the human species, allowing young women to have their reproductive function restored after treatment for oncologic diseases or other disorders that cause infertility (Dolmans et al., 2021). On the other hand, xenotransplantation (grafting between different species) is also well accepted by the scientific community as it is an excellent study model to evaluate follicular development and the resumption of ovarian function in several species (goat: (Donfack et al., 2019); sheep: (Ñaupas et al., 2021); bovine: (Kagawa et al., 2009); bitches: (Fujihara et al., 2019); non-human primate: (Yeoman et al., 2005) demonstrating that it is possible to obtain antral follicles with the potential to generate fertilizable oocytes (Wall et al., 2020).

At cryogenic temperatures, the composition of the vitrification solution, in which the biological sample is submerged inside the device is also essential to maintain follicular integrity and viability and to enable the restoration of reproductive function after cryopreservation (Lee et al., 2018).

Generally, cryopreservation solutions (freezing or vitrification) are composed of the base medium plus intra- and/or extracellular cryoprotectant agents. CPAs are organic solutes capable of protecting cells at temperatures below zero, minimizing the negative effects of low temperatures. They are considered the main components of the solution and are classified into two categories: intra and extracellular (Fuller and Paynter, 2004). Intracellular cryoprotectants have a low molecular weight (62.07 - 92.09 g/mol), high solubility, and can replace the water in the intracellular medium. The main intracellular CPAs used during ovarian cryopreservation are Ethylene glycol (EG), Dimethylsulfoxide (DMSO), Glycerol (Gly), and Propylene glycol (PRHO). On the other hand, extracellular cryoprotectants have a high molecular weight (342.29 - 504.42 g/mol) interacting with cell membrane proteins and conferring protection. The main representatives of this category are Sucrose (SAC), Trehalose (TRE), Raffinose (RAF), and the class of ice-inhibiting macromolecules such as antifreeze proteins (AFPs) and synthetic polymers (SPs) (Yong et al., 2020).

Therefore, the combination of intra- and extracellular CPAs in the vitrification solution formulation is another key factor that must be carefully defined, as the characteristics of each CPA influence the response of the biological material to the steps of the temperature reduction or warming process. In general, the main intracellular cryoprotectants that have shown positive effects on reproductive cells are glycerol (GLY), ethylene glycol (EG), dimethyl sulfoxide (DMSO), and propanediol (PROH) (El Cury-Silva et al., 2021). The combination of EG and DMSO has been the most used because the molecular characteristics of these compounds contribute to good performance in the vitrification of ovarian tissue (Carvalho et al., 2014). On the other hand, sugars, such as sucrose, act as extracellular CPAs. This type of cryoprotectant remains in the extracellular environment and increases the external osmotic pressure, resulting in cell dehydration, consequently minimizing intracellular ice formation. Furthermore, during warming, extracellular cryoprotectants also act as an osmotic buffer preventing or controlling the excessive influx of water into the intracellular medium, preventing fluid retention or cell "swelling" or even cell membrane rupture (Carvalho et al., 2013). According to the survey conducted in the present review, sucrose was the most widely used non-permeable CPAs in the consulted studies, nevertheless, other substances such as antifreeze proteins (Lee et al., 2013) and synthetic polymers have been successfully used as extracellular CPAs (Wowk, 2005).

Antifreeze proteins are originally found in living organisms that inhabit polar regions (Deller et al., 2013). The use of these proteins in the vitrification solution of ovarian tissue resulted in between 60% (mice: (Kong et al., 2018) and 70% (rabbits: (Zeng et al., 2022) and cows: (Kong et al., 2021)) of morphologically normal follicles after heating. Follicular survival was investigated, and a rate greater than 50% was observed (rabbit: (Zeng et al., 2022)). In cattle,) (Kong et al., 2021) observed for the first time the cryoprotective effect of these substances, which improves the morphology rate (70%) and reduces the follicular apoptosis level (24%).

Synthetic polymers (supercool X1000, supercool Z1000, and PVP K-12) have also gained notoriety as extracellular CPAs. These macromolecules are substances analogous to antifreeze proteins that prevent the recrystallization process (Wowk, 2005). Recent studies in goats (Vizcarra et al., 2020) and cattle (Shahsavari et al., 2020) have indicated that follicular morphology and development can be improved after in vitro culture of vitrified preantral follicles in the presence of synthetic polymers in the vitrification solution.

The studies evaluated methods and solutions used in vitro culture (80%) as a post-vitrification procedure. In humans, Ramos et al. (2022) observed that vitrified follicles showed more damage to the basement membrane than fresh follicles, while other authors did not observe this effect (Sheikhi et al., 2011; Khosravi et al., 2013; Fabbri et al., 2014; Klocke et al., 2014; Jafarabadi et al., 2015; Abir et al., 2016; Marschalek et al., 2021;). These results show that cell integrity can be preserved according to the vitrification protocol used, with data presented by (Marschalek et al., 2021) who showed the highest percentage of normal preantral follicles (83%) using a vitrification protocol containing DMSO in the vitrification solution. Although morphological damage is minimal, viability can also be a limiting factor for preantral follicle development. Shahsavari et al. (2020) demonstrated that follicular viability can be affected by vitrification, while Kagawa et al. (2009) observed no difference in the survival rate of oocytes present in the fresh and vitrified ovary (>89%). Both morphology and follicular viability are directly related to DNA molecule damage, which is extremely common during vitrification processes (Faustino et al., 2015), compromising the development and the ability to generate fertilizable oocytes (Shi et al., 2017). However, in the investigated studies, data analysis revealed that the follicles may be damaged even before being subjected to cryopreservation, showing that the quality of the ovary is also a highly relevant factor for post-cryopreservation success. After warming, it is expected that follicles will be able to fully develop and generate oocytes suitable for fertilization resulting in *in vitro* embryo production and birth of healthy individuals (Lopes et al., 2020).

## Conclusion

Not far from the rule, our observations showed that countries more economically developed in the dissemination of technological research hold more patents in the area of patents-related processes for the vitrification of ovarian tissue aimed at the preservation of the reproductive potential of the female. Consequently, this has allowed women in countries such as the United States, Japan, and Europe to have their fertility restored after removal and cryopreservation, followed by ovarian transplantation, unlike the reality found in poor or developing countries. Nevertheless, other non-patented processes can also offer the same possibility with the same safety.

Through this study, we can conclude that ovary vitrification when performed properly (device and vitrification solution), does not affect the follicular structure, preventing the endocrine and reproductive function of the female after warming. Therefore, closed devices are the most recommended, as they avoid the risk of contamination of the biological material that will be used in transplantation or in vitro culture procedures. Regarding vitrification solutions, the association of intra and extracellular CPAs is fundamental to be considered to reduce the main causes of damage during cryopreservation which are intracellular ice formation and the toxicity of intracellular CPAs. Thus, the association of EG and DMSO, as well as the use of macromolecules such as synthetic polymers and antifreeze proteins, stand out in the literature. However, standardizing an optimal vitrification protocol for ovarian tissue remains a challenge to overcome to have maximum utilization of ovarian follicular potential after warming, indicating a vast field for much future research.
